# Sociodemographic characteristics of immigrants hospitalized for first episode of psychosis

**DOI:** 10.1192/j.eurpsy.2022.1628

**Published:** 2022-09-01

**Authors:** W. Kabtni, H. El Kefi, A. Baatout, C. Bencheikh, A. Oumaya

**Affiliations:** Hmpit, Psychiatry, Tunis, Tunisia

**Keywords:** african, Tunisia, First episode of psychosis, immigrants

## Abstract

**Introduction:**

European researchers have observed that psychosis is 3 times more frequent in immigrants than in native-born subjects.

**Objectives:**

our study aims to determine the sociodemographic characteristics of immigrants hospitalized for first episode of psychosis (FEP)

**Methods:**

it’s is a descriptive retrospective study. 21 files were recruited from the psychiatry department archive. Only files of immigrant patients hospitalized, during the period between 2016 to 2021, for FEP and with neither personal nor family medical history of psychosis were included in our study.

**Results:**

A total number of 11 patients was included in our study. The analyse of sociodemographic characteristics revealed that; 62.5% of patients were female. The average age was 31 years. About half of them were dark skinned (particularly African), 25% were divorced, and 75% having university level. The majority of cases, have had a clandestine access to Tunisia, and were either unemployed or doing cleaning tasks with a low economic level and frequent conflicts in their workplaces. The average period between entering Tunisia and the onset of symptoms was 11.375 months.

**Conclusions:**

A comparative study on a larger sample would be beneficial in order to determine the risk factors for psychosis in immigrants and, consequently, leads to effective preventive measures.

**Disclosure:**

No significant relationships.

